# Sustainable biobanks: a case study for a green global bioethics

**DOI:** 10.1080/11287462.2021.1997428

**Published:** 2022-02-24

**Authors:** G. Samuel, F. Lucivero, A. M. Lucassen

**Affiliations:** a Clinical Law and Ethics at Southampton (CELS), University of Southampton, Southampton General Hospital, Southampton, UK; b Department of Global Health and Social Medicine, King’s College London, London, UK; c Ethox Centre and Wellcome Centre for Ethics and Humanities, Oxford University, Oxford, UK

**Keywords:** Digital sustainability, sustainability, environmental, biobanking, bioethics

## Abstract

This paper argues that as we move to redefine global bioethics, there is a need to be attentive to the ethical issues associated with the environmental sustainability of data and digital infrastructures in global health systems. We show that these infrastructures have thus far featured little in environmental impact discussions in the context of health, and we use a case study approach of biobanking to illustrate this. We argue that this missing discussion is problematic because biobanks have environmental impacts associated with data and digital infrastructures. We consider several ethical questions to consider these impacts: what ethical work does the concept of environmental sustainability add to the debate; how should this concept be prioritised in decision-making; and who should be responsible for doing so? We call on global bioethics to play a role in advancing this dialogue and addressing these questions.

## Introduction

The maintenance of human health and environmental degradation are closely connected (Prüss-Üstün et al., 2016; ten Have & Gordijn, 2020). Organisations who set the agenda on health policies acknowledge the importance of considering issues of environmental sustainability in order to tackle health goals.^1^ Several scholars in the field of bioethics advocate that the discipline should aim to explore the relationships between individuals and the natural environment (Dwyer, 2009; Jennings et al., 2016; Lee, 2017; Resnik, 2009; Richie, 2019). Such scholars have shown the limitations of traditional approaches to bioethics typically adopted within medical and clinical ethics, which they say tend to focus on relationships solely between individuals, and place emphasis on a narrow set of principles such as beneficence, non-maleficence, and/or individual autonomy. In contrast, these scholars advocate an “environmental turn” in bioethics, which aims to adopt a systems approach that considers individuals, populations, and environmental factors in understanding (health) practices and policies (for instance, see the special issue of (Lee, 2017), also see (Richie, 2020)). This environmental turn also aims to broaden the bioethical discussion to include issues about community, social responsibility, solidarity and justice. This endeavour has been variously labelled public health ethics, environmental health ethics, green bioethics, population ethics or global ethics, but what they all have in common is that they require the discipline of bioethics to go back to its origins, where the humanities influenced scientific developments, and the relationship between human beings and the environment was explored (Potter, 1971; Potter, 1988). Sustainability has therefore become an important concept (Richie, 2020; ten Have & Gordijn, 2020).

The wider health and other literature has focused on sustainability in different ways (Oermann & Weinert, 2016; Weaver, 2014). One example is a definition of activities continuing and enduring over time (Shearman, 1990), which is often used in healthcare when examining issues related to the implementation of specific initiatives or resource allocation (Lennox et al., 2018; Moore et al., 2017; Munthe et al., 2021). Other definitions have incorporated longitudinal, or intergenerational normative elements, such that resources continue to exist for use by others. Recent bioethics literature has drawn on this latter definition (Gurevich, 2020; Weaver, 2014) which has gained momentum over the past half a century (Yeh, 2020), and it is this definition that is the focus of this paper. In this definition, the 1987 Brundtland Commission Report defined sustainability as using resources in a way that allows resources to continue to exist for use by others (World Commission on Environment and Development, 1987). Sustainability in this reading is multifaceted, and includes the simultaneous pursuit of three interconnected development goals: economic growth, social equality/progress, and environmental protection/equilibrium. Mensah (2019) recently summarised economic sustainability as relating to the feasibility of uncontrolled growth and consumption, and requiring that decisions are made in the most equitable and fiscally sound way possible while considering the other aspects of sustainability; social sustainability as encompassing notions of equity, empowerment, accessibility, participation, cultural identity and institutional stability, and relating to the fair distribution of advantages and disadvantages within a society; and environmental sustainability as ensuring that the natural environment remains in equilibrium – productive and resilient to support human life – and that natural resources are not harvested faster than they can be regenerated, nor the waste emitted faster than what can be assimilated by the environment.^2^ This narrative of sustainability therefore embodies tensions between innovation, the equitable distribution of resources across society from one generation to the next, and consideration for the environment (Mensah, 2019; Vogt & Weber, 2019). This includes an emphasis on considering issues of global justice between different people of the present generation (intragenerational justice across people separated by space) as well as people of different generations (intergenerational justice across people separated by time (Glotzbach & Baumgartner, 2012; Kibert et al., 2012). Specifically in healthcare and in more developed countries, it has been argued that scarce resources are not being used in the most efficient way in terms of their environmental impacts (ten Have & Gordijn, 2020), and so sustainability is increasingly used as a norm that orients and informs work in this area (Callahan, 1999; Fischer, 2015; Floridi et al., 2018; Richie, 2019; Riedel, 2016; Weaver, 2014; Yeh, 2020). Nevertheless, the concept’s meaning, history, pillars and principles, and the implications of these for human development at times remain unclear. Indeed, Rolston (2002) argues that sustainable development is a “*grand policy, asserted with vigour, and then weakened with a thousand diverse applications and analyses, leaving nothing much to do in focus*”, whereas Vogt and Weber (2019) note that basic misunderstandings have led to the discourse on sustainability leading to a “dead-end” (Rolston, 2002; Oermann & Weinert, 2016; Purvis et al., 2019; Vogt & Weber, 2019).

Building on the work of scholars promoting an environmental turn in bioethics, and those who have called for a need to consider sustainability as a normative principle within the discipline, this paper brings attention to an area that has received less attention thus far – that is, the ethical issues related to the environmental sustainability of data and digital infrastructures in global health research systems that inform healthcare. Data and digital technologies coupled with artificial intelligence (AI) systems are often considered by policy makers and the industry sector as an ally in the fight against the environmental crisis and in the endeavour of sustainable development (Herweijer & Waughray, 2018; The Royal Society, 2020).^3^ Furthermore, the development and use of these technologies is quickly expanding due to their promises of saving human lives while reducing costs (Topol, 2012), as well as enabling access to health for populations living in remote areas with scarce health infrastructures (IQVIA Institute for Human Data Science, 2017). However, these accounts tend to neglect that these technologies also have negative environmental impacts that need to be counteracted. These impacts are not particularly visible in the health sector, since the laudable aim of saving lives now (or widening access to save lives now) may eclipse the potential risks to future lives through environmental damage.

Environmental impacts include the large amounts of electricity consumed to power and cool equipment in data centres – the large warehouse scale buildings where the online data that underpins the digital revolution is located (Watson et al., 2014). More widely, it has been argued that active steps need to be taken to control these environmental impacts, and meet international goals of reducing global emissions (Freitag et al., 2020). Furthermore, the extraction of minerals necessary to deliver digital technologies cause damage to biodiversity, and the manufacturing and disposal of electronic devices produces toxic emissions (Lepawsky, 2018). Although some scholars expect that efficiency improvements in digital technologies will fix the issue (The Royal Society, 2020), others expect digital pollution to grow despite improvements in efficiency of digital systems, since these improvements and the consequent lowering of costs will increase in demand and consumption (Blair, 2020; Sorrell, 2009).

In this paper, we use a case study approach of publicly funded biobanking to show how a focus on the environmental sustainability of data and digital infrastructures in a global context of health raises a range of ethical questions to be addressed. Although biobanks have been promoted in terms of public good (Busby & Martin, 2006, p. 238) the debates around biobank governance are still very immature in relation to the discussion of environmental impacts. After introducing the practice of biobanking, we demonstrate that the concept of sustainability has been used in the biobanking literature with little consideration for environmental concerns. This gap is problematic because biobanks have considerable environmental impacts that should be taken into account by discussions around their governance. In arguing for the need to focus on environmental sustainability in biobanking, we highlight a set of questions that need to be tackled: what ethical work the concept of environmental sustainability adds to the debate in relation to other concepts; how to prioritise this concept in decision-making; and who should be responsible for achieving this. We call on global bioethics communities to play a key role in addressing these questions and moving this dialogue forward.

## Biobanks and their data infrastructures

Human biobanks collect, process, store, and distribute samples, tissue and data.^4^ These biobanks – particularly those that offer large-scale research resources – promise^5^ to not only accelerate our understanding of the complex relationship between genes, epigenetic factors, and the environment in the development of disease (Caulfield et al., 2014), but also to drive precision medicine and improve global healthcare treatment and services (Chalmers et al., 2016). Much of their promised value comes from the fact that researchers do not have to expend time and funds on the collection, storage and curation of human tissue samples and data, and that by combining large-scale data sets (“big data”), researchers have greater power to answer questions of both national and global health significance (Chalmers et al., 2016). Furthermore, ancestral backgrounds may influence susceptibility to some diseases and large biobanks aim to be relevant to global ancestral populations whilst being able to describe any differences that impact healthcare. The ability of biobanks to allow many researchers to access their “big data” resource means that they are viewed as a key public benefit and are thus often funded publicly (Chalmers et al., 2016). Because of this, biobanks have expanded dramatically over the past few decades (Rush et al., 2020). Large-scale biobanks are now a mainstay in a number of countries, with some of the largest in the world being based in Europe (e.g. UK Biobank; FinnGen in Finland, Biobank Graz, Austria, The Estonian Biobank), North America (“All of us”; “CanParth”), and Asia (Biobank Japan, The Taiwan Biobank, Shanghai biobank) (Zohouri & Ghaderi, 2020).^6^ New large-scale biobanking initiatives and consortia are continuing to emerge at pace, including in Africa (54gene,^7^ H3Africa^8^), in Europe (with 21 countries signing a declaration to transnationally share data on at least 1 million human genomes by 2022 (Saunders et al., 2019)), and Asia (GenomeAsia100K^9^).

While biobanks are often associated with promissory discourses that emphasise their health benefits through breakthrough narratives (Samuel & Farsides, 2017; Tutton, 2007), scholars have raised concerns about how biobanking promotes private interests through market-based biomedical economies (Birch & Muniesa, 2020; Kahn, 2014). Specifically, worries have arisen about how value in terms of health benefit will return to the public, especially if therapies are developed with high price-tags (Sterckx et al., 2018). Alongside these concerns – and less spoken about – biobanks have an environmental impact which comes from biobank-associated data collection and processing. This is particularly the case for large-scale biobanks that collect, analyse and store large data sets, given their size and reach.^10^

The advent of biobanking’s growing popularity at the beginning of the century was accompanied by an explosion in data management and storage capabilities that are now a prominent feature of contemporary society (the “big data revolution” (Kitchin, 2014)). These advances in data collection, storage and processing allow large-scale population-based biobanks to store and process vast quantities of data. New sequencing technologies have meant that approaches to genomics are now exponentially more detailed, as well as cheaper and faster, than just a decade ago, but concomitant increases in computing power to process, analyse, store and distribute such data has a significant environmental impact (Chaterji et al., 2019). The centralisation of data through biobanks is hugely advantageous in terms of increasing the efficiency of data collection, improving data usage, and ensuring unnecessary duplication of data/data storage. At the same time, it has been predicted that by 2025, between 100 million and 2 billion human genomes will have been sequenced globally, using some 40 exabytes of data (Hogan, 2020). Over the next 5 years, the UK Biobank database – a leading biobank internationally – is expected to grow to 15 petabytes – an amount of data equivalent to that created annually by the Large Hadron Collider.^11^ However, while the environmental impact of data infrastructures is widely acknowledged in the environmental and information and communication technology literature (Code of Conduct for ICT, 2016; Environmental Protection Agency., 2007), and is considered an urgent and pressing issue (Blair, 2020; Danilak, 2017; Sorrell, 2009), these discussions have to date featured little in the biobanking literature.

## The rise of sustainability as a key concern for biobanks

As biobanks have expanded their reach, they have faced a range of ethical, social, regulatory, and economic challenges. Challenges have shifted from questions of how to collect and distribute large numbers of samples ethically; to questions of how to ensure specimens are of suitable quality for continued use; to questions about how to sustain biobanks both financially, as well as socially and operationally (Chalmers et al., 2016; Simeon-Dubach & Watson, 2014).

These questions about sustainability have exposed issues of financial sustainability: operational costs estimated at the start-up of a biobank can not keep apace with the technological advances in the volume of data created and stored mentioned above. For example, when UK Biobank recruited its cohort of 500,000 individuals, the possibility of analysing the entire genome of each participant was an unlikely speck on the horizon. Furthermore, the costs of operating expanded biobanks are now often far beyond initial expectations (Caulfield et al., 2014; Ciaburri et al., 2017; Henderson et al., 2017). Costs are incurred, for example, because of the staffing needed to procure, store, analyse and distribute a biobank resource, as well as to manage the governance of the biobank organisation; quality control costs; informatic costs; and costs related to maintaining and monitoring biosamples in freezers (Abdalijaleel et al., 2019). Sustainability issues have started to increase in scope since the establishment of many biobanks several decades ago (Doucet et al., 2017; Watson et al., 2014).^12^ A 2016 PubMed review of literature discussing issues of biobanking sustainability reported that there had been only a small number of articles published on the topic (Doucet et al., 2017); a cursory look at the literature shows a general upward trajectory of literature over time, albeit with still low numbers – from 2 articles published on the topic in 2008, to 28 articles published in 2020. The topic of sustainability has also been noted to have received specific attention at annual biobank conferences (Henderson et al., 2019), as well as featuring in several special issues of the biobanking journal *Biopreservation and Biobanking*. There are now a number of case examples in the literature of how biobanks are building sustainability into their practices (Kelly et al., 2017; Parry-Jones, 2014; Soo et al., 2017).

## Three pillars of sustainable biobanking: financial, social acceptability, and operational

While interest in biobanking sustainability has focused primarily on financial sustainability (e.g. see (Andry et al., 2017; Coppola et al., 2019; Soo et al., 2017)), over the past few years, scholars have started to recognise that (biobank) sustainability is complex, and requires a range of factors to ensure it is secured. In their 2014 paper, Watson and colleagues draw on the approach of the three pillars of sustainability described in the 1987 Brundtland report (economic, social and environmental) to provide a perspective of sustainability for the biobanking sector. Specifically, they take the pillar of economic growth and compare it with the need to ensure a biobank’s *financial* sustainability. This, they say, relates to the development and maintenance of a strategic plan through market strategy, customer focus and brand recognition. They compare social sustainability (i.e. social equality/progress) with concepts that relate to the *social acceptability* of biobanks – by the research community, the public/patient community and other non-funder stakeholders. This, they explain, includes the need for biobanks to commit to accepted standards, ethics procedures and other practices, all within an appropriate ethics governance framework. Lastly, the authors substitute the environmental protection pillar with the need to ensure a biobank’s *operational sustainability*. Operational sustainability has been defined as the aim to ensure policies and procedures improve the quality of biospecimens and increase the efficiency of biorepository operations in terms of input, internal and output components. (Parry-Jones, 2014; Seiler et al., 2015; Watson et al., 2014).

This conceptualisation of biobanking sustainability (financial, social acceptability, operational), which is also drawn upon by others (Henderson et al., 2017; Parry-Jones, 2014; van der Stijl et al., 2021), provides useful insights about biobanking sustainability. However, it is limited through the fact that this definition of sustainability, while attempting to analogise itself to the tri-pillar definition of sustainability proposed by the Brundtland Report, focusses only on *economic* sustainability. This is because the *social acceptability* and *operational sustainability* of biobanks are in fact both aspects of ensuring the economic sustainability of biobanks. This definition therefore does not draw upon environmental concerns or social sustainability (i.e. how biobanks work to address issues of global social equality and accessibility to health technologies/care that emerge from biobank-associated research). We propose that the sustainability of biobanks ought to address the entire life cycle of biobanks, including their environmental and social impact. Just as other technological developments need to, biobanks need to address the tension between innovation on the one hand, and, on the other, explicitly target their environmental and social sustainability as proposed in the Brundtland report (van Wynsberghe, 2021). In this way, the *environmental* sustainability of biobanking should then focus on questions relating to the protection of the natural environment, and *social* sustainability should focus on social justice – ensuring the equal allocation of burdens, risks, benefits, and opportunities that may come from development within all societies.^13^ By broadening the scope of how biobanking sustainability is framed, and by including the environmental and social facets of the term, it brings a perspective that allows us to take into consideration the *global* environmental and social impacts associated with biobanking.

## Environmental sustainability of biobanking: what ethical questions need to be addressed?

How could and should the lack of attention to environmental sustainability within biobanking be redressed? We note four ethical concerns.

1. How to assess (and therefore place value on) sustainability

One way to think about environmental sustainability is to simply be more explicit about this in global discourses around biobanking and highlight those aspects of the enterprise that have an environmental cost. However, given that biobanks contribute to just a tiny fraction of the environmental impacts from data and digital technologies more broadly, and there is no simple cost–benefit equation that can be used to assess what this fraction is, it makes it difficult to estimate what this cost is. This is compounded by a lack of, or inconsistent, data in the digital technology sector about the environmental impact of different processes, for example, manufacturing, supplier etc,^14^ in addition to low awareness in the biobanking sector not only about the environmental impacts of biobanks, but also around how to become more environmentally sustainable.^15^ Adopting a more specific focus on how to address environmental sustainability is unlikely to resolve these issues, because this too will require awareness and knowledge about environmental impacts that are as yet typically unknown. For example, ethical questions around whether biobank access committees should be attentive to the energy efficiency of computational methodologies that researchers use to analyse biobank data (for example, AI systems, some of which can be high energy consuming) would require researchers to be able to assess this, which at present they are unable to do because of a lack of infrastructure, processes and data at research institutions.^16^ Similar issues arise if biobanks consider the energy consumption of some of their own digital technologies. For example, some recent discussion has revolved around whether blockchain – often considered a high energy technology – could be a useful tool to assist with consent processes at biobanks (Mamo et al., 2020), but little information exists on what the environmental impact of this tool would be when compared with using other approaches (and, furthermore, how this environmental cost, if any, should be assessed against other ethical principles, such as whether this technology then improves the autonomy of biobank participants). Finally, while advances in sequencing technologies have resulted in the sequencing of entire genomes of participants (where previously only tiny fractions were analysed), it may now be appropriate to consider whether sequencing and storing genomes that are more than 99% identical in all humans holds significant advantages and whether there are environmental costs in doing so.

2. The type of sustainability we are referring to, and sustainability for *whom*?

More generally, and outside the biobanking arena, various scholars place different weight/value on each of the three pillars of sustainability (economic, social, environmental) when considering the concept, and this often depends on their discipline or interest (Vucetich & Nelson, 2010). This leads to ambiguity as to how to apply the term in any given context (Shearman, 1990). For environmental scientists, for example, sustainability is more about protecting the ecosystem, whereas engineers view sustainability as about more efficiently meeting human need (Nelson & Vucetich, 2012; Vucetich & Nelson, 2010). Furthermore, there are different types of sustainability, for example, “weak” sustainability (more in line with an economic perspective of development that considers technology and the natural environment as “capital”) and “strong” sustainability (that prioritises an environmental or ecological perspective). In addition, morally, some scholars emphasise that we should care for the environment because of its intrinsic value, while others argue that we should care for it in order to preserve resources for our children. How we view the moral justification of sustainability will have consequences. For example, if sustainability is based on an ethics of extrinsic environmental value, then other more traditional ethical principles, such as justice or inequality, might do similar work to the concept of sustainability. Finally, it seems important to ask, sustainability for *whom*? The promise of biobanks to improve the health of individuals and populations – a key united nations sustainability development goal (SDG) in itself – sits against the fact that, given societal and global structural inequalities, the benefits of this research are unlikely to meet the needs of all (for example, see (Sterckx et al., 2018)). Furthermore, it is probable that those most likely to miss out on the (promised) health benefits stemming from biobanks are also those likely to be most affected from the negative environmental impacts of the data and digital technology sector.

3. Considering how biobanks can take responsibility for being sustainable

Scholars have argued that scientists and technical professions (such as biobank personnel) have a “*heightened responsibility to assess, evaluate, and disseminate the social and environmental impacts and risks posed by technology”* because the very technologies they employ have significant impacts on the natural environment (Kibert et al., 2012, p. 70). Questions about what these responsibilities would look like in the biobanking arena require attention. For instance, in the environmental debate, people are called upon to take responsibility for their own behaviours and lifestyle choices (Ferreboeuf, 2019). At the same time, it is also highlighted that individuals alone will not solve the climate crisis if governments and industry do not align their actions to reduce emissions by addressing structural issues (Ferreboeuf, 2019). Similarly, in thinking about responsibilities of actors involved in biobanking, we need to distinguish the responsibilities that individuals (researchers, individuals governing biobanks) may have when making choices about data centre procurement, servers, and models to run to explore data, to ensure that the environmental costs are taken into account and, when possible, reduced. Alongside this, individual choices are constrained by the larger ecosystem (funders, professional bodies, university management) that they are part of and that need to create incentives to promote environmentally friendly choices in this context (Samuel & Lucivero, 2020). For example, there may be no further resources or funding to allow the most environmentally appropriate choice to be made in terms of assessing and measuring environmental impacts, procurement and energy supplier decisions and/or choices related to data centre providers. This is especially the case if funders, university management and/or research institutions do not act responsibly to provide such funding. As such, while biobanks may want to act responsibly, if the network to do so does not exist, and if the responsibility to do so is not enacted across the whole ecosystem, it makes it increasingly difficult for this desire to be enacted (Samuel et al., 2021; Samuel & Lucivero, 2020).

4. Considering what a sustainability ethical framework would look like in practice

Riedel (2016) argued that we need an ethical framework to help “*clarify the ethical founding principles, which normative facets, which practice-relevant and decision-making values and moral duties are inherent in sustainability, and which, in their turn, could form a basis for decision-making”*. Here other scholarly work in the health arena has already made a start on trying to define such principles and frameworks relevant for guiding practice. For example, Riedel (2016) argued that the term sustainability should be associated with three moral norms and values in nursing practice: “*justice (distributive justice or fairness, questions about the distribution of health-relevant goods and services); responsibility (professional, moral and causal responsibility); and quality of life (subjective and objective assessments of quality of life)*” (Riedel, 2016). Similarly, in her work on green bioethics, Christie proposes four normative principles to guide environmentally sustainable healthcare; distributive justice, resource conservation, simplicity, ethical economics (Richie, 2019).

However, these values and principles can be weighted in different ways, according to specific contexts (Riedel, 2016), and this brings tensions and quandaries to decision-making– for example relating to the relationship between intragenerational and intergenerational justice (Glotzbach & Baumgartner, 2012).^17^ Furthermore, they need to be balanced against other values associated with society and the economy (Kuhlman & Farrington, 2010).^18^ As Seiler and colleagues (2015) astutely note in their examination of biobanking sustainability more generally, the challenge is not in defining the pillars of sustainability, but in prioritising these concepts within the context of biobanking’s broader organisation goals (Seiler et al., 2015).

## Conclusion: from biobanking to global bioethics

The increasing use of digital technologies and the interconnection between human health and environmental wellbeing make the issue of ensuring that this digitalisation of the health sector does not endanger the environment and the health of inhabitants, even more compelling. In this paper, we have used the example of biobanking to bring a sustainability gaze to the bioethics and health field (Richie, 2019; Riedel, 2016). We have raised a series of questions that the biobanking sector will need to take seriously if they are to consider environmental sustainability within their endeavours and we consider these questions will also need to be addressed by the health sector more broadly. We argue that there is a need for a reframing and rethinking of global bioethics to include such questions related to the unintended environmental impacts associated with the use of data and digital technologies. We call on a global bioethics to take on this role moving forward.

## Notes

1 See, for example, actions on environment and health by the World Health Organisation, the Wellcome Trust (https://wellcome.org/what-we-do/our-work/our-planet-our-health), or the National Institute for Health (https://www.niehs.nih.gov/).

2 More recently concerns have been raised about the clear distinction between the three pillars, with sustainability scholars moving towards the application of a more integrated approach with cross-section terms Fischer, M. (2015). “Fit for the Future? A New Approach in the Debate about What Makes Healthcare Systems Really Sustainable.” *Sustainability* **7**(1).,

3 Also see https://itu.foleon.com/itu/aiforgood2019/home/.

4 The term “biobank” is a heterogeneous concept, and can be applied to a range of different institutions and initiatives. This complexity has arisen from an increasingly diverse set of biobank purposes, and of types and sources of the samples; different biobanks may vary from simple to very complex structures, and may exist for a short time or be maintained for many years. Simeon-Dubach, D. and P. Watson (2014). “Biobanking 3.0: evidence based and customer focused biobanking.” *Clin Biochem* **47**(4–5): 300-308.

5 Jurisdictions have worked hard to sell the promise of biobanks to their societies, with discourses around biobanks being associated with, for example, nationalism Busby, H. and P. Martin (2006). “Biobanks, national identity and imagined communities: The case of UK biobank.” *Science as Culture* **15**(3): 237-251.

6 Also see https://www.biobanking.com/10-largest-biobanks-in-the-world/.

7 https://54gene.com/.

8 https://h3africa.org/.

9 https://genomeasia100k.org/.

10 Not the focus of this paper, but they also have an environmental impact from the materials used during the biosampling handling processes, as well as from the energy required to store such samples. For example, freezers required to house biosamples need a significant amount of energy to work (the lower the temperature the more energy required). These freezers also need to be housed in temperature-controlled rooms, which require energy to be maintained, and need to be replaced on a regular basis (every decade or so) meaning issues relating to waste disposal https://biobankinguk.org/environmental-sustainability-in-biobanking/; https://www.ucl.ac.uk/sustainable/case-studies/2019/mar/sustainable-biobanking.

11 https://www.ukbiobank.ac.uk/learn-more-about-uk-biobank/news/uk-biobank-creates-cloud-based-health-data-analysis-platform-to-unleash-the-imaginations-of-the-world-s-best-scientific-minds.

12 Perhaps because of a lack of foresight, or perhaps because biobanks could not have predicted the explosion of “big data”

13 We also emphasise the need to consider social sustainability. This is not the focus of this paper, though we note the interrelation of environmental and social sustainability issues in terms of social justice and inequality. Global bioethics can also have a role in exploring these issues.

14 Findings from interviews conducted by the authors with stakeholders working in the digital sustainability sector; manuscript in draft format.

15 Findings come from a small survey of European biobanks conducted by the authors; manuscript in draft format.

16 Some scholars are working to categorise AI systems using sustainability development goals (presentations at the Sustainable AI conference, June 15-17, 2021. Germany (and online). https://www.sustainable-ai.eu/.

17 Kilbert and colleagues ask, “how is it possible to address the needs of future people when the needs of the vast majority of the world’s present population are not being met? What exactly are the ‘needs’ that must be met?” Kibert, C. J., L. Thiele, A. Peterson and M. Monroe (2012). The Ethics of Sustainability.

18 Sometimes the three pillars of sustainability may align or facilitate each other, in other instances they may be in direct tension.
